# Evaluating supervised and unsupervised background noise correction in human gut microbiome data

**DOI:** 10.1371/journal.pcbi.1009838

**Published:** 2022-02-07

**Authors:** Leah Briscoe, Brunilda Balliu, Sriram Sankararaman, Eran Halperin, Nandita R. Garud

**Affiliations:** 1 Bioinformatics Interdepartmental Program, University of California Los Angeles, Los Angeles, California, United States of America; 2 Department of Computational Medicine, David Geffen School of Medicine, University of California Los Angeles, Los Angeles, California, United States of America; 3 Department of Computer Science, University of California Los Angeles, Los Angeles, California, United States of America; 4 Department of Human Genetics, David Geffen School of Medicine, University of California Los Angeles, Los Angeles, California, United States of America; 5 Department of Computational Medicine, David Geffen School of Medicine, University of California Los Angeles, Los Angeles, California, United States of America; 6 Department of Anesthesiology and Perioperative Medicine, David Geffen School of Medicine, University of California Los Angeles, Los Angeles, California, United States of America; 7 Institute of Precision Health, University of California Los Angeles, Los Angeles, California, United States of America; 8 Department of Ecology and Evolutionary Biology, University of California Los Angeles, Los Angeles, California, United States of America; University of Trento, ITALY

## Abstract

The ability to predict human phenotypes and identify biomarkers of disease from metagenomic data is crucial for the development of therapeutics for microbiome-associated diseases. However, metagenomic data is commonly affected by technical variables unrelated to the phenotype of interest, such as sequencing protocol, which can make it difficult to predict phenotype and find biomarkers of disease. Supervised methods to correct for background noise, originally designed for gene expression and RNA-seq data, are commonly applied to microbiome data but may be limited because they cannot account for unmeasured sources of variation. Unsupervised approaches address this issue, but current methods are limited because they are ill-equipped to deal with the unique aspects of microbiome data, which is compositional, highly skewed, and sparse. We perform a comparative analysis of the ability of different denoising transformations in combination with supervised correction methods as well as an unsupervised principal component correction approach that is presently used in other domains but has not been applied to microbiome data to date. We find that the unsupervised principal component correction approach has comparable ability in reducing false discovery of biomarkers as the supervised approaches, with the added benefit of not needing to know the sources of variation apriori. However, in prediction tasks, it appears to only improve prediction when technical variables contribute to the majority of variance in the data. As new and larger metagenomic datasets become increasingly available, background noise correction will become essential for generating reproducible microbiome analyses.

## Author summary

The human gut microbiome is known to play a major role in health and is associated with many diseases including colorectal cancer, obesity, and diabetes. The prediction of host phenotypes and identification of biomarkers of disease is essential for harnessing the therapeutic potential of the microbiome. However, many metagenomic datasets are affected by technical variables that introduce unwanted variation that can confound the ability to predict phenotypes and identify biomarkers. Currently, supervised methods originally designed for gene expression and RNA-seq data are commonly applied to microbiome data for correction of background noise, but they are limited in that they cannot correct for unmeasured sources of variation. Unsupervised approaches address this issue, but current methods are limited because they are ill-equipped to deal with the unique aspects of microbiome data, which is compositional, highly skewed, and sparse. We perform a comparative analysis of the ability of different denoising transformations in combination with supervised correction methods as well as an unsupervised principal component correction approach and find that all correction approaches reduce false positives for biomarker discovery. In the task of predicting phenotypes, different approaches have varying success where the unsupervised correction can improve prediction when technical variables contribute to the majority of variance in the data. As new and larger metagenomic datasets become increasingly available, background noise correction will become essential for generating reproducible microbiome analyses.

## Introduction

The human gut microbiome is associated with a number of host phenotypes including colorectal cancer [1], obesity [2,3], and antibiotic consumption [4–7], among other traits [8,9]. Despite the promise of the microbiome as a diagnostic tool, significant challenges remain for predicting phenotypes and finding reproducible biomarkers of human phenotypes from microbiome data. One major challenge is that technical covariates, including sample storage [10], cell lysis protocol [11,12], extraction method [13,14], DNA preservation and storage protocol [15], preparation kit [16,17], and primer choice [11], are known to introduce unwanted variation and systematically bias the relative abundances of taxonomic features in microbiome samples [12,18–24].

These covariates, when differentially distributed across phenotypes, can act as confounders. There are two potential outcomes of confounding in prediction accuracy: increased accuracy when confounders are consistently correlated with the phenotype, or decreased prediction accuracy when the confounder is oppositely correlated with phenotype from one subset of the data to another. In either scenario, confounding is problematic for detecting true associations between the microbiome and phenotype. The pooling of datasets is a major contributor of confounding yet combining datasets is an increasingly common [1,25–28] and powerful means to validate associations [8,29] in a discovery dataset with held out datasets [1,30,31]. Recent studies have shown that confounding covariates are widespread in genomic datasets. Gibbons *et al*. [32] found that combining datasets to detect members of the microbiome that are associated with colorectal cancer resulted in false positive detection of differentially abundant taxa. Confounding covariates were also pervasive [33] in one of the largest metagenomic datasets available, the American Gut Project (AGP) [34].

Despite the widespread effects of background noise in microbiome data, there is currently a dearth of methods specially equipped for removing unwanted variation in microbiome data. Initial steps in processing microbiome data often involve addressing differences in library sizes across samples by applying the variance-stabilizing transformation (VST) from DESeq2 [35] or the log_2_-counts per million (logCPM) from EdgeR [36] on taxonomic counts data [37–42]. However these transformations do not sufficiently address other contributors of unwanted variance such as study-specific covariates, which neccessatates explicit methods for correction. Existing methods repurposed from other domains for this purpose, including gene expression^39,40^ and methylation [43–45], generally fall into two categories: supervised methods, where the sources of variation must be explicitly specified, and unsupervised methods, where the sources of variation are first inferred and then removed before association or prediction analyses. The most popular supervised methods are batch mean centering (BMC)^43^, which centers data batch by batch, and ComBat^44^ and limma^45^, which both use empirical Bayes. Many studies will apply a supervised method after applying one of the above transformations in microbiome data. However, since many sources of variation may be unknown, and moreover, the extent of variation they introduce may vary from dataset to dataset [20,32,46–48], unsupervised approaches [49–51] for covariate correction may be more effective in removing background noise. Among the unsupervised approaches are ReFactor [51], Surrogate Variable Analysis (SVA) [49], and Remove Unwanted Variation (RUV) [50] which were designed for methylation or gene expression data. These methods quantify “surrogate variables” that represent study-specific effects and regress them out of the data.

Despite their promise, the repurposed supervised and unsupervised approaches [49–51] are not suitable for microbiome data because most of them rely on assumptions that the data is normally distributed. However, taxonomic features are often sparse [52,53] due to taxa having abundances below the detection limit of sequencing [52], or taxa being absent in certain samples, resulting in skewed non-normal distributions. Additionally, because the microbiome data is usually transformed into measures of relative abundances, the data is compositional, or in other words, represented as relative frequencies of taxonomic features within a sample that sum to one. This representation also causes non-normal distributions.

Supervised methods proposed explicitly for microbiome data to reduce background noise include percentile normalization [27], Partial Least Squares Discriminant Analysis [54], and multiplicative bias correction [22]. Both percentile normalization [27] and Partial Least Squares Discriminant Analysis [54] aim to find predictive features in fully labeled data with known batches and known phenotypes, and are not designed for prediction of phenotypes in unlabeled data, while multiplicative bias correction [22] requires either a reference sample in which the species abundance distribution is known or a term specifying the experiment label, and thus cannot account for multiple sources of background noise simultaneously. Given that these methods are supervised and thus cannot be applied to unlabeled data, there still remains a need in the microbiome field for unsupervised approaches that can adjust for both measured and unmeasured variables. Additionally, there is little published research comparing adapted approaches head-to-head in microbiome data.

To address the need for unsupervised approaches applicable to microbiome data, we examined a popular approach used in the field of population genetics known as Principal Components Analysis (PCA) correction. Population structure is often strongly reflected in the first principal components (PCs) calculated from genotype data [55–57]. By removing the effect of the first few PCs in a regression approach, association testing can be done to find potential genetic biomarkers of phenotype rather than biomarkers of population structure [55–57]. PCA correction has been effective in correcting for confounding covariates in human genetic data [55,57] and morphological data[58], but to date has not been applied to microbiome data. Yet, we and others find that top principal components in multiple datasets are correlated with numerous confounding variables like host genetics [59], ethnicity of the host [60], and also abiotic factors like temperature [61], suggesting that PCA correction may be an effective unsupervised correction approach.

In this paper, we evaluated the ability of PCA correction to remove background noise in microbiome data and compared its performance to supervised background noise correction approaches [62–64] that are commonly used for microbiome data. Specifically, we tested the impact of regressing out principal components (PCs) from microbiome data featurized as abundances of taxonomic features or *k*-mers. Abundance of taxonomic units are determined by aligning or binning reads based on reference genomes, whereas *k*-mer abundances are calculated by counting appearances of short substrings of length *k* in raw sequences. While taxonomic features have immediate biological interpretability, the use of *k-*mers is beneficial because they do not rely on a reference genome. Additionally, we assess the impact of applying a variance stabilizing transformation (VST) or logCPM (log counts per million), and compare this to application of the centered log ratio (CLR). CLR is more widely used for compositional data, particularly in microbiome contexts [29,65–70], and is a suggested transformation prior to factor analysis such as PCA because it breaks the dependence between features [66] and makes data more normally distributed [54]. This transformation can make the PCs more interpretable because the transformed value is the abundance relative to the mean value for a sample.

By performing a comparative analysis of PCA correction and existing supervised correction approaches, we evaluate the merits of repurposing the PCA correction approach from the field of population genetics to the microbiome, as well as assess the strengths and limitations of various methods. Throughout this study, we highlight important considerations for phenotype association studies from large cohort and cross-study metagenomic analyses, which we hope paves the way for higher reproducibility across microbiome studies.

## Results

We analyzed four metagenomic datasets for evidence of technical covariates that could introduce noise or confounding that, as a result, interfere with biomarker discovery and prediction accuracy. We evaluated the ability of three popular supervised approaches for microbiome data (ComBat [64], limma [63], and batch mean centering (BMC) [62]), three transformations (CLR, VST from DESeq2 [35] and logCPM from EdgeR [36]), and an unsupervised approach, PCA correction, to correct for noise and confounding. We focused on three phenotypes of interest: body mass index (BMI), colorectal cancer (CRC), and antibiotic consumption (Table 1). The datasets we analyzed included: (i) the American Gut Project [34] (AGP), which has known confounding variables [33], (ii) a pooled dataset composed of three 16S datasets with healthy and CRC individuals (hereafter referred to as ‘CRC-16S’) [27], (iii) a pooled dataset composed of seven whole metagenome sequenced datasets (WGS) with healthy and CRC individuals (hereafter referred to as ‘CRC-WGS’) [1,71], and (iv) the Hispanic Community Health Study (HCHS) [72] consisting of 16S samples from over one thousand individuals from several Hispanic countries. These datasets allowed us to assess noise and confounding both within datasets (AGP and HCHS) and across pooled datasets (CRC-16S and CRC-WGS).

**Table 1:** Datasets used in this study. Two pooled datasets composed of multiple studies are abbreviated as CRC-16S [73–75] and CRC-WGS [1,74,76–79], whereas the American Gut Project (AGP) [34] and the Hispanic Community Health Study (HCHS) [72] are each from a single source study and have several potential confounders [33].

Phenotype	Joined dataset	Number of samples	Number of studies	Sequencing method	Published Sources
Body mass index	American Gut Project (AGP)	6,722	1 (multiple sequencing batches)	16S	[34]
Antibiotic history	American Gut Project (AGP)	12,619	1 (multiple sequencing batches)	16S	[34]
Body mass index	Hispanic Community Health Study (HCHS)	1,769	1 (multiple sequencing batches)	16S	[72]
Colorectal Cancer	CRC-16S	574	3	16S	[73–75]
Colorectal Cancer	CRC-WGS	813	7	WGS	[1,74,76–79]

### Background noise detected by principal component analysis

**Fig 1:**
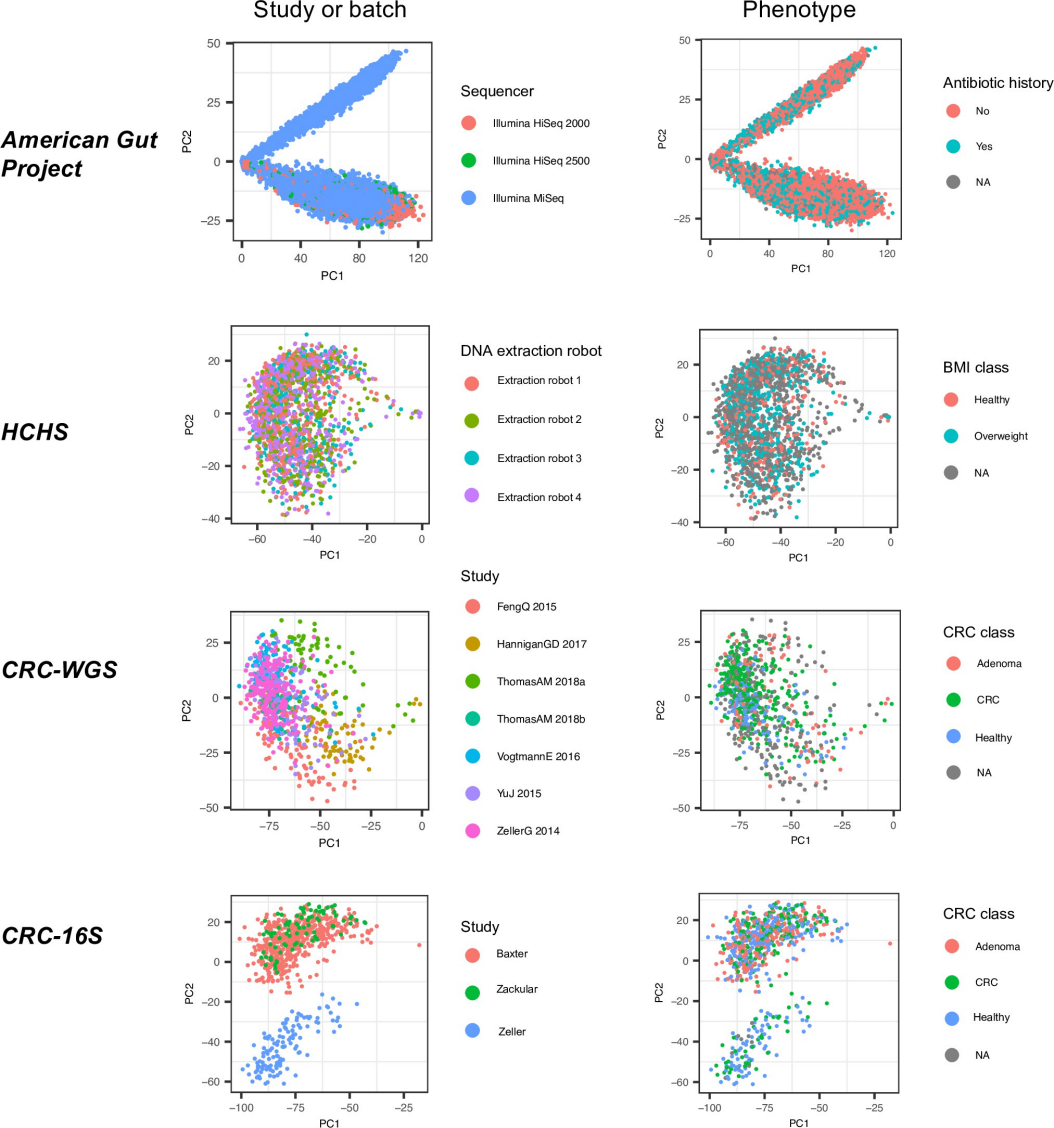
First two principal components of across datasets. PCA applied to CLR-transformed taxonomic abundance data from the four datasets of the study. Each point represents a single microbiome sample colored by either study or batch and by phenotype group.

To assess the extent of microbiome variation attributable to technical covariates, we performed PCA on CLR-transformed (see Methods) taxonomic abundance profiles and short *k-*mers (between sizes 5 and 8) derived from the raw metagenomic reads (see Methods). In most cases, for the first two PCs, samples cluster by dataset and not the primary phenotype of interest (Figs 1 and S1), consistent with previous findings [13] that technical factors have a strong effect on the microbiome.

**Fig 2:**
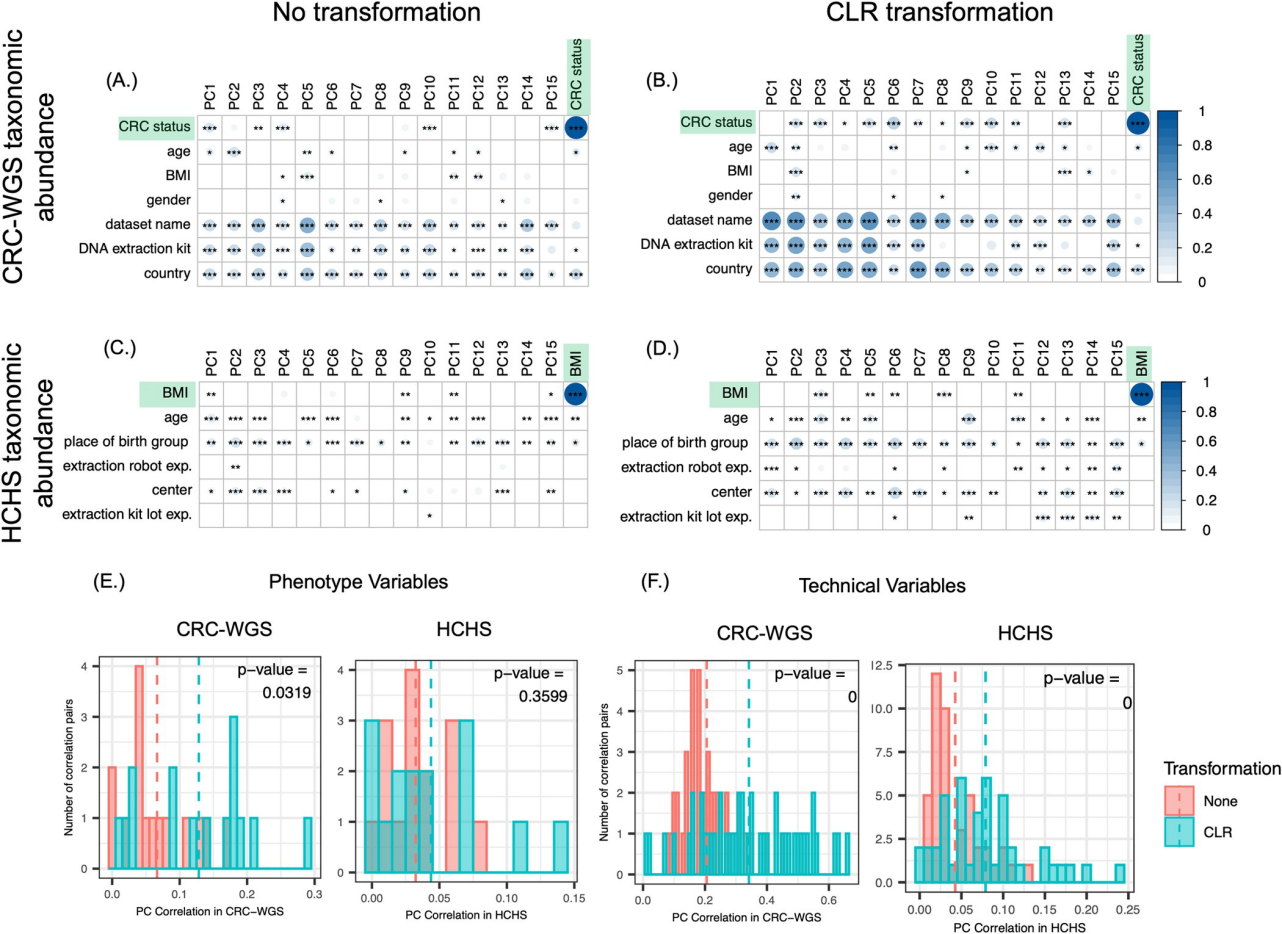
Microbiome data is affected by technical and biological variables. **(A-D)** Heatmaps of canonical correlations between the first 15 PCs and study covariates in CRC-WGS with (A) no transformation and (B) after CLR transformation; and in HCHS with (C) no transformation and (D) after CLR transformation. **(E,F)** Histograms of the correlations in (A-D) where the distributions were compared using a paired Wilcoxon signed-rank test to test whether the distribution of correlations from PCs of CLR-transformed are greater than the untransformed. The size and color of the circles in each cell in A-D indicate the magnitude of correlation and black asterisks indicate the significance of the Pearson correlation of the PCs with each of the variables. The color bar at right represents the range of correlations observed across all datasets. [*,**,*** indicate Wilcoxon signed-rank *p*-values as follows: 10^−2^ < *p* < 0.05, 10^−3^ < *p* < 10^−2^, *p* < 10^−3^]. See S2 and S5 Figs for similar analyses for the other datasets, and S6 Fig for other transformations.

More generally, the top 15 PCs in each dataset are more correlated with technical variables than the phenotypes of interest (Figs 2A and S2). For example, in the CRC-WGS dataset, PCs one through five on average have a 0.28 mean correlation with dataset label but only 0.072 mean correlation with CRC status (Fig 2A). It is worth noting that these first five PCs collectively explain 84% of the variance in the CRC-WGS data and that the strongest correlations with CRC status are in the first five PCs. In the HCHS dataset, the top 5 PCs have significant correlation with demographic information such as place of birth (0.13 mean correlation of top 5 PCs) and sequencing center (0.09 mean correlation of top 5 PCs), but only a mean 0.04 correlation with BMI. In this dataset, the first five PCs collectively explain 59% of the variance in the HCHS dataset but only the first PC is significantly correlated with BMI, where PC1 explains 24% of variance (Fig 2D).

We next assessed the impact of CLR-transformation on the correlation of top PCs with technical and biological covariates, and compare the correlations using a two-sample Wilcoxon signed-rank test. Firstly, across all datasets, CLR-transformation of taxonomic abundance and *k*-mer data results in more normally distributed data (**S3 Fig**), making the data more suitable for PCA. However, the change in correlation of the top PCs with technical and biological covariates after application of the CLR transformation varies from dataset to dataset. In the case of both AGP and CRC-WGS datasets, the CLR transformation results in siginificantly increased correlation of the top PCs with both biological and technical covariates (Figs 2A and 2B and S4) (in the CRC-WGS dataset, median correlation of PCs with CRC increased from 0.05 to 0.14 with Wilcoxon signed-rank p-value = 0.03 and median correlation with technical covariates increased from 0.19 to 0.32 with Wilcoxon signed-rank p-value < 2.22 x 10^−3^; in the AGP dataset, median correlation of PCs with BMI and antibiotic history increased from 0.16 to 0.31 with Wilcoxon signed-rank p-value = 1 x 10^−4^ and median correlation with technical covariates increased from 0.05 to 0.07 with Wilcoxon signed-rank p-value = 8.7 x 10^−3^) (Fig 2B and 2C). In the CRC-16S dataset, neither biological or technical variates showed significantly increased correlation after CLR transformation variables (CRC median correlation increased from 0.05 to 0.10 with Wilcoxon signed-rank p-value 0.084; technical covariate median correlation changed from 0.09 to 0.08 with Wilcoxon signed-rank p-value 0.12). Unlike all the other datasets, application of the CLR transformation to the taxonomic abundances of the HCHS dataset results in a significantly increased correlation with technical variables, but not biological variables (BMI median correlation increased from 0.029 to 0.033 with Wilcoxon signed-rank p-value = 0.36; technical covariate mean correlation increased from 0.03 to 0.07 with Wilcoxon signed-rank p-value < 2.22 x 10^−3^) (Fig 2E and 2F). These correlations are all the more striking given the high percentage of variance explained by the first five PCs alone: 80% of variance in the CRC-WGS dataset, 64% of variance in the AGP dataset, and 65% of variance in the HCHS dataset. We similarly assessed the impact of logCPM and VST transformations on the correaltions of the top 15 PCs with technical and biological variables in S6 Fig and found that correlations with study covariates also increase.

We also assessed the impact of *k*-merization on the correlation of variables with top PCs. Unlike for taxonomic abundances, CRC-WGS does not show significant change as a result of CLR transformation on *k*-mers (S5 Fig). In the AGP dataset, median correlations with BMI and antibiotic history increase from 0.55 to 0.57 (Wilcoxon signed-rank p-value = 8 x 10^−4^), and in the HCHS and CRC-16S datasets, correlations with technical variables increase from a median of 0.04 to 0.07 (Wilcoxon signed-rank p-value = 0.001) and a median of 0.1117 to 0.1125 (Wilcoxon signed-rank p-value = 0.0498) after the CLR transformation. Through these analyses on taxonomic abundance and *k*-mers, we show that technical variables introduce considerable variation in microbiome data sets, that this variation is often larger than variation explained by phenotypes of interest. Transformations like CLR can additionally make this variation explained by technical variables more apparent.

### Reduction of false positive biomarker discovery as a metric of background noise correction

**Fig 3:**
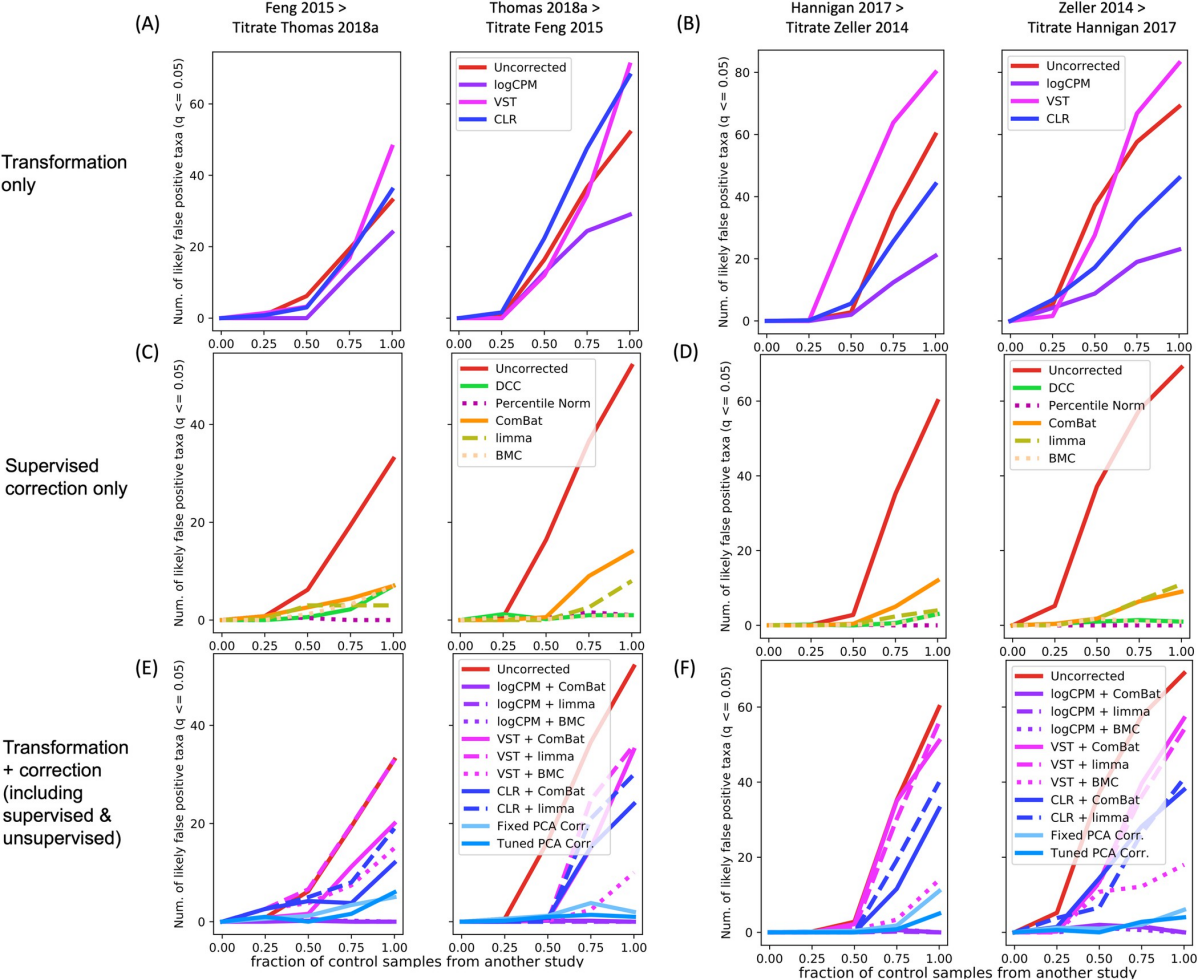
Spurious association of taxa with case-control status without appropriate correction. (A) We tested the number of associations identified after replacing the controls from the CRC-WGS study sequenced by [1] referred to as Thomas et al. 2018a with controls from Feng et al. at increasing proportions and vice versa. (B) Similarly, controls in the CRC-WGS study Hannigan et al. [79] were replaced with controls from Zeller et al.[74] and vice versa (S7 Fig). BMC + CLR was an outlier and excluded for clarity of visualization, but the summary of mean associations of BMC + CLR is in S1 Table.

Pooling of datasets is frequently done to augment power to detect associations with or make predictions about host phenotype [1,25,26,28,71]. However, this practice can also result in false positive associations due to confounding between study-specific variables and phenotype [27]. Thus, we tested the ability of different background noise correction methods to reduce false positive biomarker discoveries. To do so, we performed a titration experiment similar to that described in Gibbons et al. [32] in which control groups from two different studies in the CRC-WGS dataset were mixed at different proportions to create a new control group of equal size that was then compared with cases to identify taxa significantly associated with disease using a Wilcoxon rank sum test with false discovery rate correction (q-value < 5%). Without correction, spurious associations are expected to increase with increasing proporion of control samples coming from a different study (Fig 3). We compare correction approaches by ascertaining the number of likely false positive associations at different titration levels (proportions of control samples from another study) ranging from 0% to 100%. In the scenario where 100% of controls are from a second study, the study variable is a complete confounder for case-control status.

To asses the efficacy of transformations to reduce false positive associations, we first compared the untransformed and uncorrected relative abundance data to each of three data denoising transformations: logCPM, VST and CLR applied to feature counts. As expected, when the data is untransformed, the number of new taxa identified that are likely false positives steadily increases as the number of control samples added from a second study increases, reaching 42 when 100% of controls are from the second study. When the data is transformed with logCPM, VST, or CLR, the number of likely false positives reaches 20, 52, and 44, respectively (Figs 3A and 3B and S7 and S1 Table), indication that transformation alone does not always reduce false positives.

Next, we assessed the ability of supervised background noise correction methods to suppress false positives. These methods included percentile normalization [27], BMC [62], ComBat [64], and limma [63] which require a batch variable to be specified. Thus, in these cases we corrected for the variables that are the most correlated with the top PCs in each dataset: sequencing instrument in the AGP dataset, processing robot in the HCHS dataset, and source study in the CRC dataset. We additionally included a supervised correction approach in which these same primary contributors of heterogeneity were directly regressed out, an approach we term in this paper as Direct Covariate Correction (DCC) (see Methods). When 100% of controls are from the second study, the number of likely false positives drops to 5, 0, 5, and 6 respectively for the DCC, percentile normalization, ComBat, and BMC methods (Figs 3C and 3D and S7 and S1 Table).

Next, we evaluated the effectiveness of applying the logCPM, VST, and CLR tranformations in combination with the supervised approaches ComBat, limma, and BMC (Fig 3E and 3F), a practice which is currently done in the literature for microbiome studies [37–42]. We also compared these approaches to two variants of unsupervised correction in which PCA correction is applied after CLR: one in which the optimal number of top PCs are identified via cross-validation and regressed out from the data and another in which data is corrected for a fixed and arbitrary number of PCs. We refer to these two variants as tuned PCA and fixed PCA, respectively (see Methods). Tuned PCA uses a validation set to determine the optimal number of PCs that maximize prediction accuracy while fixed PCA correction corrects for the first three PCs (Methods). The choice of three PCs for this analysis was arbitrarily selected to avoid completely throwing away the signal associated with the phenotype of interest.

When 100% of controls are from the second study, logCPM applied prior to ComBat, limma, or BMC results in 1, 2, and 2 likely false positive associations, respectively (Figs 3D and 3E and S7 and S1 Table). When the VST transformation is applied prior to ComBat, limma, or BMC, we find 45, 55, and 25 likely false positive associations (Figs 3D and 3E and S7 and S1 Table). When the CLR transformation is applied prior to ComBat, limma, or BMC, we find 26, 35, and 173 likely false positive associations (Figs 3D and 3E and S7 and S1 Table). Lastly, when Fixed PCA and Tuned PCA is applied along with CLR, we find 14 and 11 likely false positive associations, respectively.

Overall, these results suggest that data transformations should not be applied alone and that a transformation like logCPM can be applied before applying a supervised correction in order to reduce the appearance of false positive associations. Alternatively, unsupervised approaches where CLR is applied prior to PCA correction can also reduce false positive associations.

### Cross-study prediction after background noise correction

A successful predictive model is transferable across datasets. To assess the impact of background noise correction on phenotype prediction, we performed a leave-one-dataset-out (LODO) analysis. For this analysis, we utilized a nested cross-validation scheme where one dataset was set aside for testing of a prediction model that was trained and validated on the remaining datasets using either a Random Forest classifier or linear regression model (see Methods). We evaluated the impact of supervised and unsupervised background noise correction approaches, with and without data transformations, on prediction of host phenotype using taxonomic abundance profiles and *k*-mers (see Methods), where binary phenotype prediction accuracy is assessed by Area Under the Curve (AUC) and continuous phenotype prediction accuracy is assessed by Pearson correlation.

**Fig 4:**
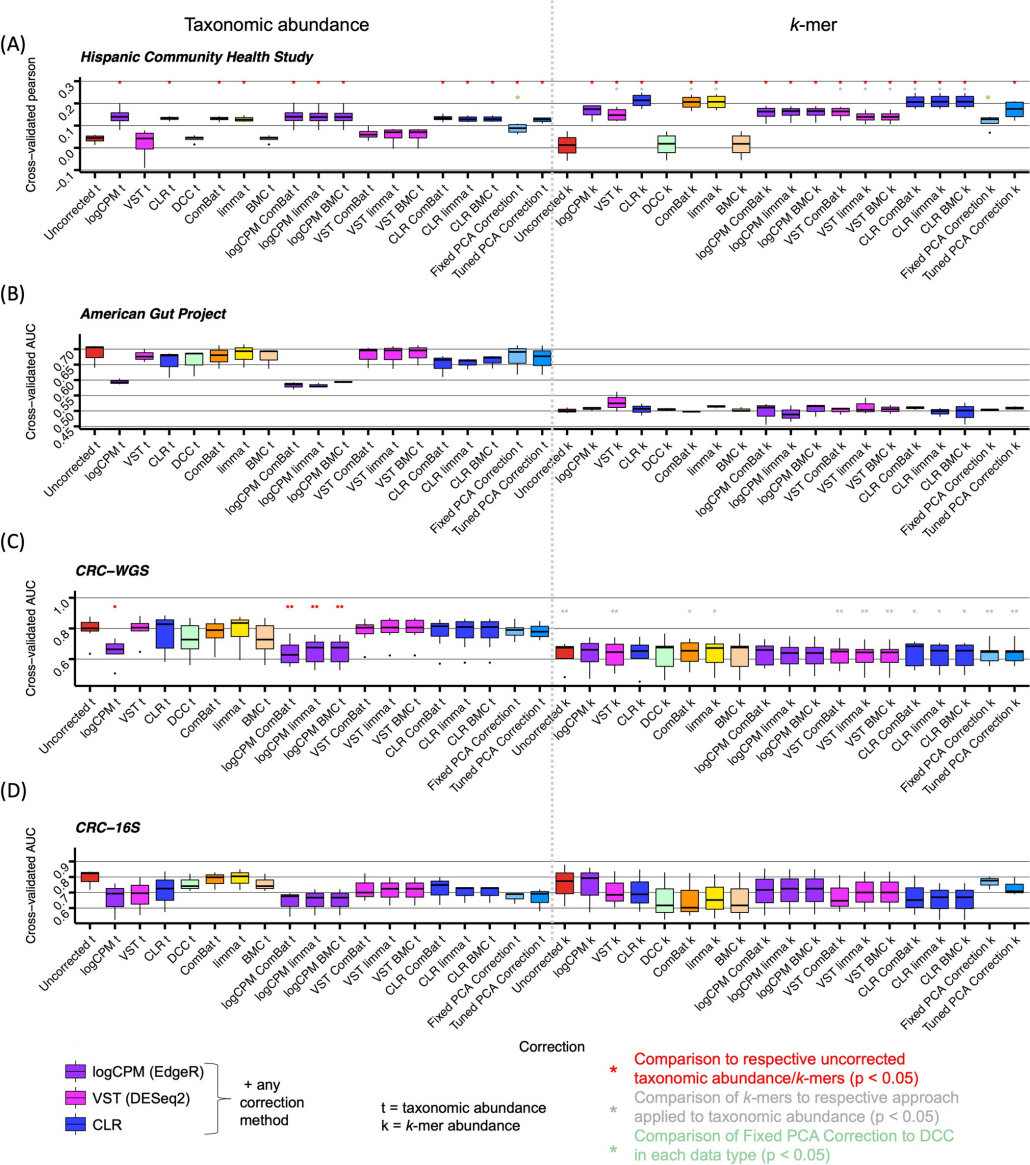
Phenotype prediction models generalize across studies after application of noise correction methods. Cross-study prediction of **(A)** body mass index (BMI) in the HCHS dataset across different extraction robots **(B)** antibiotic consumption in the past year in the AGP dataset across different Illumina sequencing models, **(C)** CRC status in the CRC-WGS dataset across different studies and **(D)** CRC status in the CRC-16S dataset across different studies. The boxplots in (A) indicate leave-one-dataset-out Pearson correlation between true and predicted BMI, for each batch. (B-D) indicate leave-one-dataset-out AUC for each held-out study or batch. p-values comparing each boxplot were computed using a one-sided Wilcoxon signed-rank test. A red * indicates a significant difference in prediction ability compared to uncorrected data in the respective taxonomic or *k*-mer group. A grey * indicates a significant difference in prediction between the *k*-mer (k) and taxonomic abundance (t) groups for a given approach. A green * indicates a significant difference in prediction between the Fixed PCA correction and DCC for a given data type. Due to the low number of folds in LODO prediction (3 to 7 values per box plot), many tests did not yield a p-value.

We first compared the effect of the different transformation and corrections on prediction of BMI, a continuous phenotype. When applying a transformation only to taxonomic abundances, logCPM and CLR resulted in significantly better Pearson correlations between the true and predicted BMI (0.04 under uncorrected increased to 0.14 and 0.13 median Pearson across batches with one-sided Wilcoxon rank-sum p-value = 0.014 for both), but VST did not show any significant improvement (one-sided Wilcoxon rank-sum p-value = 0.443) (Figs 4A and S8). When applying supervised correction approaches without transformations to taxonomic abundance data, we found that ComBat and limma significantly improved prediction to 0.13 median Pearson (one-sided Wilcoxon rank-sum p-value = 0.014 for both) while DCC and BMC did not (one-sided Wilcoxon rank-sum p-value = 0.557). Finally, applying a transformation followed by supervised correction, logCPM or CLR followed by ComBat, limma, or BMC resulted in significantly improved prediction (one-sided Wilcoxon rank-sum p-value = 0.014 for all). Applying Fixed or Tuned PCA correction, which includes a CLR transformation prior to regressing on PCs, also significantly improves prediction (one-sided Wilcoxon rank-sum p-value = 0.014 for both). Because DCC is the only method that explicitly adjusts for primary confounders, we also compared Fixed PCA correction directly to DCC and found that Fixed PCA is significantly better than DCC with median Pearson increasing from 0.045 to 0.089 (one-sided Wilcoxon rank-sum p-value = 0.014) suggesting that unsupervised correction may more broadly correct for noise that interferes with BMI prediction.

We next assessed the prediction performance using *k*-mers instead of taxonomic abundances. Uncorrected *k*-mer abundances have worse prediction accuracy than taxonomic abundances. However, when *k*-mer abundances are transformed with logCPM, CLR, ComBat or limma alone, or a combination of VST or CLR with a supervised correction, the prediction improves significantly compared to using taxonomic abundance with the highest median Pearson of 0.21 resulting from applying CLR alone (one-sided Wilcoxon rank-sum p-value = 0.014) (Figs 4A and S8). In particular, the use of *k*-mers with a CLR transformation and any correction method, supervised or unsupervised, surpasses prediction accuracy using taxonomic abundance. CLR combined with supervised correction results in a median Pearson correlation of 0.21 and CLR combined with Tuned PCA correction results in a median correlation of 0.17 (one-sided Wilcoxon rank-sum p-value = 0.014 for comparison with uncorrected *k*-mers) (Figs 4A and S8). As with taxonomic abundance, Fixed PCA is significantly better than DCC applied to *k*-mers with median Pearson increasing from 0.018 to 0.13 (one-sided Wilcoxon rank-sum p-value = 0.029).

Next, we evaluated prediction ability with two binary phenotypes: whether an individual had consumed an antibiotic in the previous year and whether an individual has been diagnosed with colorectal cancer (CRC). For the taxonomic abundance profiles of the AGP, CRC-WGS and CRC-16S datasets, applying a data transformation alone did not significantly change the AUC results with the exception of logCPM in the CRC-WGS dataset where accuracy decreased significantly (median AUC went from 0.80 to 0.66, one-sided Wilcoxon rank-sum p-value = 0.0055) (Figs 4B–4D and S8). Applying any supervised correction method by itself or after a data transformation did not result in any change in prediction ability, except when logCPM was applied with any supervised correction method to the CRC-WGS dataset, resulting in decreased accuracy (median AUC went from 0.80 in uncorrected to 0.76 for all supervised methods, one-sided Wilcoxon rank-sum p-value = 2 x 10^−3^, 3.5 x 10^−3^, 2 x 10^−3^ for ComBat, limma, BMC) (Figs 4C and S8).

Unlike for the BMI phenotype, *k-*mers showed significantly lower prediction accuracy than taxonomic abundances irrespective of correction method in the CRC-WGS and AGP datasets (Figs 4C and S8). Fixed and Tuned PCA correction on *k-*mers were able to maintain prediction accuracy of uncorrected *k-*mers for all three binary phenotype datasets (Figs 4B–4D and S8). For the CRC-16S dataset, application of both data transformation and correction methods to *k*-mer abundances resulted in increased accuracy of CRC prediction, but there is insufficient data to find significant increases, with both PCA corrections resulting in the highest accuracy (Fig 4D). The benefit of utilizing *k*-mers is most apparent in predicting BMI in HCHS, whereas in other datasets, taxonomic abundance data is better. These results indicate that for some phenotypes, correction can improve prediction accuracy, and in most cases accuracy is at least maintained.

## Discussion

The ability to predict human phenotypes from metagenomic data is important for the discovery of biomarkers of disease and the subsequent development of therapeutics. However, a major issue that impacts prediction and biomarker discovery is the presence of confounders and systemic background noise both within [33] and across studies [22,27]. In this paper, we investigated the ability of different denoising transformations in combination with supervised correction methods to correct for sources of background noise in microbiome data and evaluated the utility of an unsupervised approach–PCA correction on CLR-transformed data. We recognize that fully correcting for background noise and population-specific factors, particularly in an unsupervised manner, is extremely difficult if not impossible. Further, biological variables associated with population-specific factors can be helpful for prediction of phenotype and applying correction approaches can potentially remove the effect of these variables. For that reason, we do not advocate for one approach over the other, but instead we highlight the issues that can arise when study-specific effects are not appropriately accounted for and demonstrate several approaches to combat these effects.

In this study, we analyze four datasets: AGP, HCHS, CRC-WGS, and CRC-16S. The AGP and HCHS datasets provided the opportunity to evaluate intra-study heterogeneity, whereas the CRC-WGS and CRC-16S datasets provided the opportunity to evaluate inter-study heterogeneity. These are particularly unique datasets because they are either very large (AGP and HCHS), or they are comprised of several datasets measuring the same phenotype (CRC-WGS and CRC-16S), which is uncommon. For example, our decision to focus on CRC-WGS was motivated by important findings in Wirbel et al. [71] and Thomas et al. [1], two studies which compiled a collection of metagenomic samples from healthy and CRC individuals across a total of seven cohorts. Both these studies were able to find a core set of CRC-associated microbes despite differences in ethnicity, diets, and other host factors across studies. Both Wirbel et al. [71] and Thomas et al. [1] found that CRC classification models generalized effectively across studies and reported similar mean LODO AUCs of 0.81. We were able to also predict CRC with a similar accuracy of AUC 0.79 both before and after correction. In addition to CRC, we found prediction of BMI to be a useful analysis because it is notoriously difficult to predict accurately [80–82].

Given the diverse range of datasets available, there is not one data denoising transformation or correction method that outperforms the others universally, and multiple methods should be tested for phenotype analysis. This motivated a broad comparison of popular transformations and correction approaches. PCA correction has been effective in correcting for unwanted variation in human genetic data and morphological data [55–58], but to date has not been evaluated for correction of such noise in microbiome data. Yet, we and others have shown that top principal components in multiple datasets are correlated with numerous potential sources of unwanted noise such as host genetics [59], ethnicity of the host [60], and also abiotic factors like temperature [61], suggesting that PCA correction may be an effective unsupervised correction approach. We found that regressing out the top PCs after applying a CLR transformation may address multiple issues simultaneously: first, this approach can prevent inflation of false positives associations (Fig 3), second, can maintain and, in the case of BMI, increase prediction accuracy of host-associated phenotypes in a LODO analysis (Fig 4).

Our comparison of correlations between PCs and study covariates sheds light on which datasets are good candidates for PCA correction. In the HCHS dataset, where PCA correction was most successful, correlation of technical covariates and not biological covariates with the top PCs increased significantly after CLR transformation (Figs 2 and S2 and S4). This potentially allowed for removal of technical noise without sacrificing phenotype signal, perhaps even enhancing the phenotype signal. The result was that application of CLR along with any correction method to both taxonomic abundances and *k*-mers was successful in increasing prediction accuracy (Fig 4A). On the other hand, the CRC-WGS and AGP datasets had an increased correlation of both biological and technical covariates with the top PCs after CLR transformation (S4 Fig), making the removal of technical noise without removing phenotypic signal difficult. In these cases, applying any transformation or correction approach did not improve accuracy and instead in most cases resulted in similar performance to uncorrected data. Thus, the extent of background noise differs from one dataset to another, and the success of an unsupervised versus supervised method varies for each dataset (Table 2).

**Table 2:** Key considerations when performing background noise correction in metagenomic data.

**Taxonomic features**	***K*-mer features**
• Pro: Find directly interpretable biomarkers of phenotype • Pro: May be better for prediction of binary phenotypes like colorectal cancer • Con: features are often rare, resulting in a sparse feature matrix unless features we are grouped to genus or family level	• Pro: Not reliant on reference genomes • Con: Features not immediately interpretable • Pro: May be better for prediction of certain continuous phenotypes like BMI • Pro: Short *k*-mer sizes are more Gaussian distributed and non-sparse
**No transformation of features**	**CLR transformation of features**
• Pro: Useful for compositional analysis. Sufficient when feature distribution meets assumptions regarding normality • Con: Compositional data does not meet assumptions of many types of differential abundance analyses.	• Pro: Useful to apply to compositional data before PCA for interpretability [93] • Pro: Produces a Gaussian-like distribution (log transformation may also accomplish this) • Con: May be problematic for correlation-based analyses [94] • Note: Other transformations (edgeR and DESeq2) may be useful
**Supervised Correction**	**Unsupervised Correction**
• Pro: Correction is targeted and most influential batch effects are explicitly accounted for • Con: Need metadata on experimental setup (batches or study-effect groups)	• Pro: Do not need information on batches or study-effect groups, but helpful for assessing signal of study effects • Pro: Multiple sources of noise can be corrected for simultaneously • Con: Correction is less targeted and biological signal may be sacrificed.

Despite correction approaches having limited effect on prediction ability for most datasets, these same correction approaches had a large impact on reducing false positive biomarker associations in our titration analysis. Specifically, we found that when performing association analyses, a supervised correction applied after a denoising transformation may be best and that transformations alone are insufficient to reduce false positive discoveries (Fig 3).

In this work, we show that CLR has comparable ability to other denoising transformations both when used alone and in combination with other correction approaches. The application of CLR transformation can address many attributes of microbiome data that make it difficult to model including sparsity and non-normality, which existing unsupervised approaches designed for non-microbiome data [49–51] are ill-equipped to deal with (Table 2). As PCA assumes features are normally distributed, we produced Q-Q plots (S3 Fig) showing that the quantiles of CLR-transformed data are close to the quantiles of a theoretical normal distribution. The application of CLR to microbiome data has been broadly recommended [66,83] and is part of a suite of methods known as Compositional Data Analysis (CoDA) [84,85] to address the dependency between features inherent to compositional data. However, the adoption of CLR in the microbiome field has not been uniform. Recently, McLaren at al. [22] discussed that CoDA methods’ ability to make microbiome data invariant to multiplicative bias has been underappreciated within the field. Specifically, McLaren et al. [22] found that that ratio-based analyses could remove intra-study bias, though did not address its effect on multiple datasets that are pooled together or large datasets with heterogeneous sampling procedures such as the AGP. Here, we provide the first systematic investigation into the effect of how CLR in combination with PCA can remove inter-study and intra-study bias. We hypothesized that applying CLR transformation will more readily reveal the covariates that introduce technical background noise across and within heterogeneous datasets because these contributors of bias (e.g. DNA extraction method, sequencing instrument, etc.) have a multiplicative effect on relative abundances [22]. We found that indeed relationships between the microbiome and such variables is more apparent after CLR transformation, our observation of this in taxa abundance profiles makes sense in the context of multiplicative bias expounded by McLaren et al. [22] because the multiplicative bias becomes additive in log space, such that PCA is able to capture the bias in the top PCs as a shift in the centroid of samples plotted for a given dataset (Fig 2). Just as we found CLR transformation can significantly effect PC correlations with covariates, the application of data transformations like variance-stabilizing from DESeq2 [35] and the log counts-per-million (logCPM) transformation from EdgeR [36] applied to taxonomic abundance also affect the correlation of variables with top PCs (S6 Fig). Simiarly, these transformations can be helpful for phenotype prediction (Figs 4 and S8).

We also compared the impacts of correction on *k-*mers and taxonomic features (Table 2). *K-*mers are a useful way to featurize data because they are not dependent on reference genomes. Moreover, short *k-*mers of size 5–8 have the added benefit of a Gaussian-like distribution (S5 Fig) and low sparsity, unlike taxonomic features. However, *k*-mers have inherent limitations because they are usually not directly interpretable biological features. This limitation may be a reason why taxonomic feature abundance outperforms *k-*mers in phenotype prediction accuracy (Figs 4 and S8). It is crucial to note however, that *k*-mers may provide a better signature of technical artifacts like PCR bias [86,87] and are also known to be protocol specific [88]. Thus, this may explain why for both 16S and WGS data, *k*-mers had higher correlations with technical variables compared to taxonomic features (Figs 2 and S2 and S5). This aspect of *k-*mers offers a potential explanation for why PCA correction was particularly effective with *k*-mers for the HCHS dataset. Of note, these correlation analyses may reveal associations between linear effects of PCs and covariates, but not for non-linear effects. Other have also found that *k*-mers performed poorly compared to counts of reads aligned to reference genomes [89]. In predicting CRC and antibiotic consumption status, species profiles were more predictive whereas in predicting BMI, *k*-mers were more predictive under the majority of correction approaches when compared to application of the same approach to taxonomic abundance.

The supervised approaches [62–64] are beneficial in that they directly remove known confounding, potentially at the cost of phenotype prediction, while unsupervised approaches are can correct for both measured and unmeasured factors of microbiome (Table 2). Correcting for confounders and PCs both can result in the removal of phenotype signal, as is the case in ComBat [64] and fixed PCA (Figs 4 and S8). Tuned PCA may reduce the removal of phenotypic variance by removing up to, but not including, the first PC that would significantly impact phenotype signal. However, caution must be taken when using tuned PCA in the presence of strong confounding as it may not remove all confounding to protect the phenotype effect. In these scenarios, one should consider either a liberal correction of confounding by correcting for more PCs or subsampling the data such that cases and controls are matched for known confounders as is done in Vujkovic-Cvijin et al. [33].

Background noise correction is becoming increasingly important as the microbiome field matures and new datasets become available. One exciting future application of correction that we foresee is in microbiome wide association studies in which microbiome genomic polymorphisms are associated with human phenotypes [90,91]. Such a scenario may benefit from background noise correction since population structure may play a considerable confounding role [92]. As researchers consider the best approach for background noise correction for their specific research questions, they must weigh the tradeoffs between addressing confounding while also maintaining as much of the phenotype signal as possible. There is no single solution that will address all problems, but at minimum researchers should perform careful forensics to investigate the nature and pervasiveness of confounders in their data. In this manner, consistent and robust inferences can be made across multiple studies, moving us towards the goal of accurate phenotype prediction from microbiome data.

## Methods

### Datasets

Raw 16S fastq files were downloaded from the NCBI Sequence Read Archive (SRA) with study accessions PRJEB11419 for the American Gut Project, and PRJNA290926 [73] and PRJEB6070 [74] for CRC-16S. Fastq files for [75] from CRC-16S were obtained from http://mothur.org/MicrobiomeBiomarkerCRC/. The raw WGS fastq files for CRC-WGS were downloaded from SRA with study accessions PRJEB12449 [78], PRJEB10878 [77], PRJEB7774 [76], PRJNA447983 [1], PRJEB6070 [74], and PRJNA389927 [79]. Processed OTU data for the AGP was obtained from Qiita study id 10317 (EBI submission ERP012803). OTU profiles from CRC-16S were obtained from the MicrobiomeHD database [8]. Taxonomic profiles for CRC-WGS were obtained through the R package curatedMetagenomicData [30] which used MetaPhlAn2 [95]. In both MicrobiomeHD and curatedMetagenomicsData, taxonomic abundances were computed in the same pipeline for each set of studies.

### *k*-mer processing

Features in metagenomic data can be defined in two broad ways, both high-dimensional: reference-based approaches and reference-free approaches. Reference-based approaches cluster sequenced reads based on a defined threshold and assign taxonomy by aligning reads to reference genomes. Reference-free approaches, sort reads into bins that are defined independently of known genomes, i.e. *k*-mers, short strings of length *k* that can be obtained directly from read sequences, which are increasingly popular in microbiome data analyses and have been used by several studies to do prediction. *K*-mers offer a powerful alternative approach to more commonly used taxonomic features, because they do not rely on a reference database of genomes and do not require identifying a set of parameters to determine taxonomic features.

To compute *k*-mer abundances, raw sequences from either 16S or whole metagenome sequencing were input into the *k*-mer counting algorithm Jellyfish 2.3.0[96] with default parameters except for a hash of 10 million elements and canonical *k*-mers with size of 5, 6, 7 or 8. Prior work has shown that *k*-mer sizes of 6 and 7 are predictive of phenotype[69]. The resulting *k-*mer abundance table is then converted to a composition such that each sample sums to 1 to account for different reads depths across samples. Taxonomic profiles were similarly converted to compositions.

### Centered log ratio transformation

The centered log ratio (CLR) transformation is a compositional data transformation that takes the log ratio of between observed frequencies and their geometric means. This is done within each sample where relative frequencies of different taxa are measured and sum to 1. This can be written in mathematical form as:

$$clr(x)=\;\left[\log\frac{x_{1}}{G(x)},\log\frac{x_{2}}{G(x)},\ldots ,\log\frac{x_{n}}{G(x)}\right]=[logx_{1}–log\;G(x),logx_{2}–log\;G(x),\ldots ,logx_{n}–log\;G(x)]$$

$$G(x)={\left(\prod_{i=1}^{N}x_{i}\right)}^{1/N}$$

Here, ***x*** is a vector representing the abundance of microbiome features in a single sample, and *G*(***x***) represents the geometric mean. The Gaussian-like distribution of CLR-transformed microbiome compositional data is shown in S3 Fig. We added a pseudocount equal to 0.65 times the minimum non-zero relative abundance, following zero-replacement strategies as suggested by, prior to applying the CLR transformation.

### Background noise correction methods

The existing supervised approaches for background noise correction compared in this study include percentile normalization[32], batch mean centering (BMC)[62], ComBat[64], and limma[63] applied to relative abundance data. ComBat[64] assumes data is cleaned and normalized prior to batch effect removal. We added a pseudocount equal to 0.65 times the minimum non-zero relative abundance, following zero-replacement strategies as suggested by. It’s common to add a pseudocount to 0 relative abundance observations so that one can apply a log transform in the normalization prior to ComBat[64] (as described in Gibbons et al.[27]). We followed this same procedure with both OTU and *k*-mer, and applied ComBat[64] and limma[63] to the log of relative abundance data. For percentile normalization, batch mean centering (BMC), and Direct Covariate Correction (DCC) we used the relative abundance.

For phenotype prediction and titration analysis, a relative-abundance feature is needed. ComBat, limma, and PCA corrected data will often produce non-positive data that does not resemble counts. To create count-like data we took the exponent of the resulting ComBat and limma corrected data produces count like features.

The CLR transformation and PCA-Correction used the relative abundance of *k*-mers and taxonomic features. The equation used to regress out confounding covariates in DCC is as follows:

$$X^{m\times n}∼\beta^{m\times b}C^{b\times n}+\epsilon^{m\times n}$$

Where the original feature matrix *X* with *m* features and *n* samples is the outcome of a linear model with covariate associated coefficient matrix *β*, dummy matrix *C* with each row representing one of the *b* possible values of the confounding covariate, and *ϵ*, the residual matrix. The residual matrix *ϵ* is the covariate-corrected feature matrix. To perform titration and downstream prediction analysis on PCA-corrected data, we performed an inverse-clr as implemented in the compositions R package to convert data to relative abundance.

In PCA correction, top PCs computed from the CLR transformed *k*-mer or OTU relative abundance tables are regressed out. The CLR transformation cancels out the multiplicative bias within each study by taking a ratio of features to the geometric mean of features that are all impacted by the same study-specific multiplicative bias. The transformation accentuates the difference in bias across studies by smoothing out the intra-study bias, thereby allowing PC regression to account for the confounding across studies. In the fixed PCA correction, a set number of PCs are regressed out from the microbiome data. In the main figures we show results after regressing up to three PCs. Alternatively, the tuned PCA correction uses a train-validation-test approach to tune two hyperparameters: the optimal number of PCs to regress out *p*, and, when using *k*-mers, the optimal *k*. The same portion of data used for validation in the Random Forest tuning is used for tuning the PCA correction hyperparameters, where the tuned Random Forest hyperparameters are fixed before tuning *p* and *k*. To determine the number of PCs that optimize phenotype prediction, PCs 1 through *p* were regressed out of the input data with *p* ranging from 1 to 20. The *p* that produces the highest AUC or Pearson correlation in phenotype prediction (method of prediction model described below) in validation was selected. The same procedure is done with *k* where values between 5 and 8 are tested (only *k*-mer sizes 6 and 7 were tested for CRC-WGS) The reported performance is based on the remaining 20% set aside for testing.

### Correlation analyses

To compute the correlation of PCs with covariates before and after CLR correction, we used canonical correlation analysis using the ‘canCorPairs’ function in the R package variancePartition[97]. We used canonical correlation because several covariates were categorical, with the result that only positive correlation values can be calculated. The distribution of correlations before and after CLR transformation were statistically compared using the two-sample Wilcoxon signed-rank test.

### Phenotype prediction

In CRC-16S and CRC-WGS, we predicted whether a sample comes from a host with colorectal cancer or a healthy host. For the American Gut Project, we predicted whether a sample comes from a host who took antibiotics in the previous year or a host who has not taken antibiotics in the previous year. We also use the American Gut Project to predict body mass index (BMI).

We performed prediction of binary traits using Random Forest implemented in Scikit-learn [98], which has been previously employed successfully for predicting binary outcomes from microbiome data [1,30,99,100]. We tuned four hyper-parameters of the Random Forest model in a grid search using a train-validation-test strategy. In the LODO framework, one study was reserved for testing while the remaining studies were split such that 70% of samples were used for training and 30% for validation of model hyper-parameters. In the non-LODO framework, 56% of samples in the meta-cohort were used for training, 24% for validation of model hyper-parameters, and 20% reserved for testing, where the distribution of studies or sub-cohorts were similar in the test, train, and validation sets. Six hyperparameters where four were tuned in a grid search: estimator trees (100, 1000, or 1500), criterion (entropy only), minimum samples per split (2, 5, or 10), minimum samples per leaf (1, 5, or 10). Two hyperparameters were trained using the following settings: max depth of trees was set at ‘None’ (nodes are expanded until all leaves contain only one class or until all leaves contain less than min_samples_split samples [98]) and maximum features was set to “auto” (set to square root of number estimator trees [98]), and default parameters otherwise. This was performed in five-fold cross validation repeated ten-times to obtain confidence intervals on the area under the ROC curve (AUC), our metric of prediction accuracy. A similar train-validation-test strategy was used for the linear regression model to select coefficients of the model where accuracy was measured using Pearson correlation of the true BMI to the predicted BMI. The difference in the distribution of prediction accuracy for both prediction tasks was quantified statistically using a Wilcoxon rank-sum test.

### Titration

Following the procedure from Gibbons et al. [32], samples from different studies were pooled together to assess the inflation of false positive associations. The minimum class membership across two studies was used as the set sample size drawn from the case and controls for each study for a given titration experiment. A fraction of 0, 25, 50, and 100% controls in the first study were replaced with controls from a second study. The filtering of features as implemented in Gibbons et al. required features resembling relative abundance, and we therefore, applied the appropriate transformations to convert ComBat, limma, and PCA-corrected data to relative abundance. For ComBat and limma, we applied the natural exponent of the matrix. For CLR-transformed data (including PCA-corrected data), we applied the ‘inverse clr transform’ as implemented in the ‘compositions’ package in R [70].

## Supporting information

S1 Fig First two principal components from microbiome dataset studied. PCA was applied to taxonomic abundance profiles and 6-mer data from the AGP, CRC-WGS merged dataset, CRC-16S merge datasets, and Hispanic Community Health Cohort. Samples were plotted along the first 2 PCs with colors indicating (A) dataset or batch membership and (B) phenotype label.
(TIF)

S2 Fig Top principal components from the CRC-16S dataset correlate with technical and biological covariates. The first 15 PCs in the CRC-16S taxonomic abundance joined datasets are correlated with variables measured in each of the studies, including phenotype, sex, age, race, dataset label, sequencing method, library size and several others in (A, B) AGP, (C, D) CRC-16S. The size and color of the circles in each cell indicate the magnitude of correlation while black asterisks indicate the significance of the Pearson correlation of the PCs with each of the variables. The color bar at right of each plot represents the range of correlations observed across all datasets. [*,**,*** indicate *p*-values as follows: 10^−2^ < *p* < 0.05, 10^−3^ < *p* < 10^−2^, *p* < 10^−3^].
(TIF)

S3 Fig Quantile-Quantiles plot for AGP, CRC-WGS, and CRC-16S before and after the CLR-transformation. The quantiles of 100 randomly-selected taxonomic features or *k*-mers, that were converted to z-scores, ranked against the expected quantiles from a normal distribution of mean 0 and variance 1. The R-squared values are reported in the annotated text.
(TIF)

S4 Fig Histogram of correlation between top 15 PCs and various measured variables. Histograms show the distribution of correlation values computed between the top 15 PCs of taxonomic features in each dataset and the phenotype covariates and technical covariates. Shown in black text are the Kolmogorov-Smirnov test p-values for the test of the null hypothesis that the distribution of correlations in the non-transformed data is no different from the correlations in the CLR-transformed data. HCHS is the only dataset with significant increase in correlation in the technical covariates but not the phenotype of interest.
(TIF)

S5 Fig Top principal components from 6-mers correlate with technical and biological covariates. The first 15 PCs before (a, c, e, and g) and after (b, d, f, and h) the CLR-transformation are correlated with variables measured in each of the studies, including dataset label, library size, DNA extraction kit used, country of origin, age, body mass index (BMI), sex, and colorectal cancer status (CRC). The size and color of the circles in each cell indicate the magnitude of correlation while black asterisks indicate the significance of the Pearson correlation of the PCs with each of the variables. The color bar at right of each plot represents the range of correlations observed across all datasets. [*,**,*** indicate *p*-values as follows: 10^−2^ < *p* < 0.05, 10^−3^ < *p* < 10^−2^, *p* < 10^−3^].
(TIF)

S6 Fig Top principal components from LogCPM and VST transformed taxonomic abundance correlate with technical and biological covariates. The first 15 PCs from data transformed with the (A) EdgeR log counts per million (LogCPM) transformation[36] and (B) DESeq2 Variance Stabilizing (VS) transformation are correlated with variables measured in each of the studies, including dataset label, library size, DNA extraction kit used, country of origin, age, body mass index (BMI), sex, and colorectal cancer status (CRC). The size and color of the circles in each cell indicate the magnitude of correlation while black asterisks indicate the significance of the Pearson correlation of the PCs with each of the variables. The color bar at right of each plot represents the range of correlations observed across all datasets. [*,**,*** indicate *p*-values as follows: 10^−2^ < *p* < 0.05, 10^−3^ < *p* < 10^−2^, *p* < 10^−3^].
(TIF)

S7 Fig Titration analysis for new false positive associations. For each study in CRC-WGS, an equal number of cases and controls were drawn to determine significant taxa associated with CRC. Then, at proportions of 25%, 50% and 100%, control samples were replaced with controls from a second study. This experiment was repeated after applying (A) transformations, (B) corrections, or (C) a combination of both (including unsupervised methods) to compare the extent to which new false positive associations arise with increasing confounding between CRC and study label.
(TIF)

S8 Fig Impact of correction approaches on phenotype prediction accuracy. Heatmap showing AUC or Pearson correlation in a cross-validated prediction model using either uncorrected data or data after applying one of the following covariate correction approaches: DCC, ComBat[64], limma[63], BMC[62], and Fixed PCA correction with three PCs regressed out, and Tuned PCA correction where the number of PCs regressed out is a tuned hyperparameter. The testing accuracy mean shown is obtained from a five-fold cross validation repeated 10 times.
(TIF)

S1 Table Mean number of new associations in titration experiment. Shown is the mean number of likely false positive associations with respect to the original study 1 case and controls before adding control samples from study two, across all pairs of studies within CRC-WGS and across all five-fold replicates of titration at each mixing proportion of 0%, 25%, 50%, 75%, and 100% controls from study two.
(DOCX)
