# Supplementary figures and images for: Evaluating supervised and unsupervised background noise correction in human gut microbiome data
Source: PLoS Comput Biol. 2022 Feb 7;18(2):e1009838. doi: 10.1371/journal.pcbi.1009838 (PMC8853548; doi:10.1371/journal.pcbi.1009838)

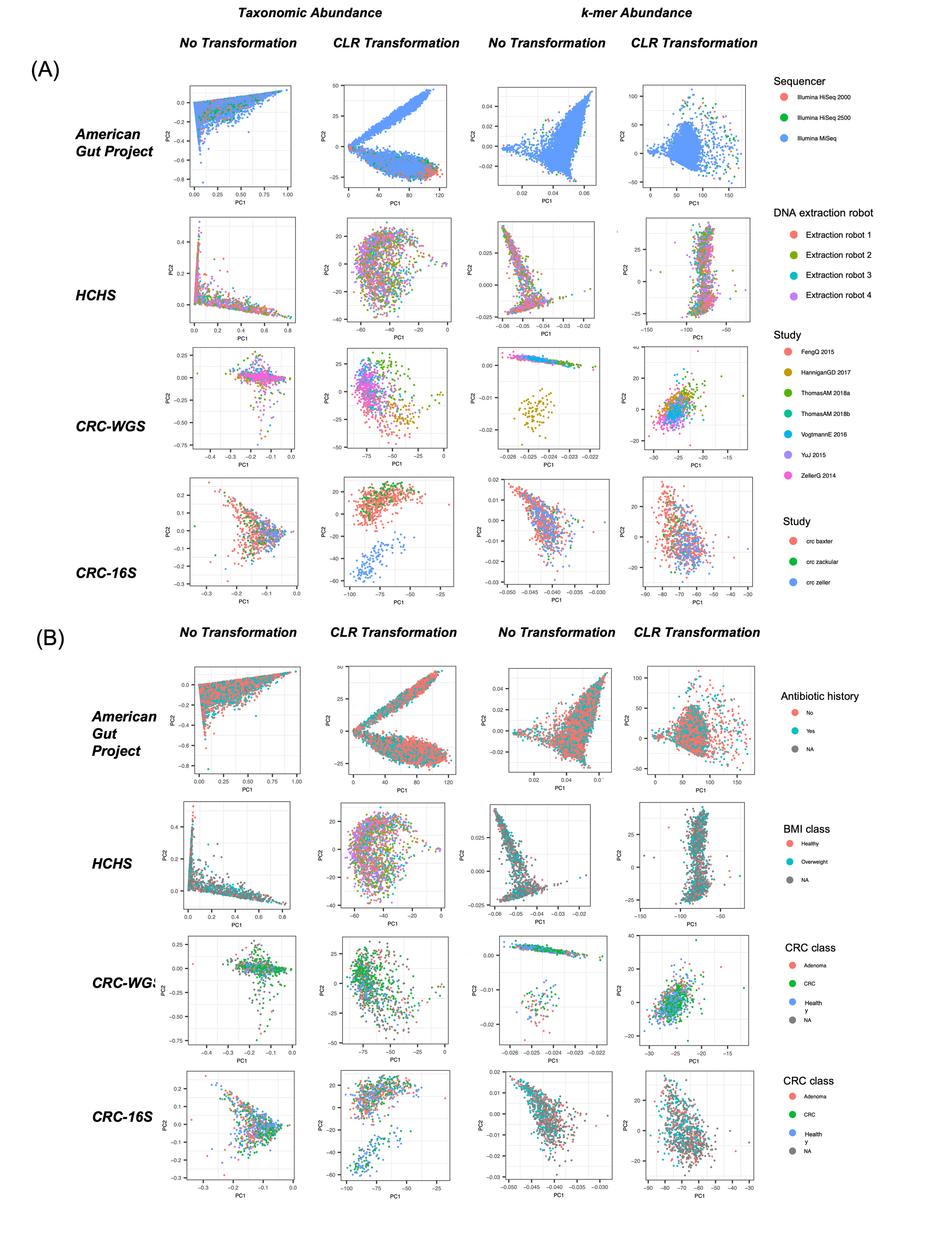

Supplement: S1 Fig — PCA was applied to taxonomic abundance profiles and 6-mer data from the AGP, CRC-WGS merged dataset, CRC-16S merge datasets, and Hispanic Community Health Cohort. Samples were plotted along the first 2 PCs with colors indicating (A) dataset or batch membership and (B) phenotype label. (TIF) [file pcbi.1009838.s001.tif]

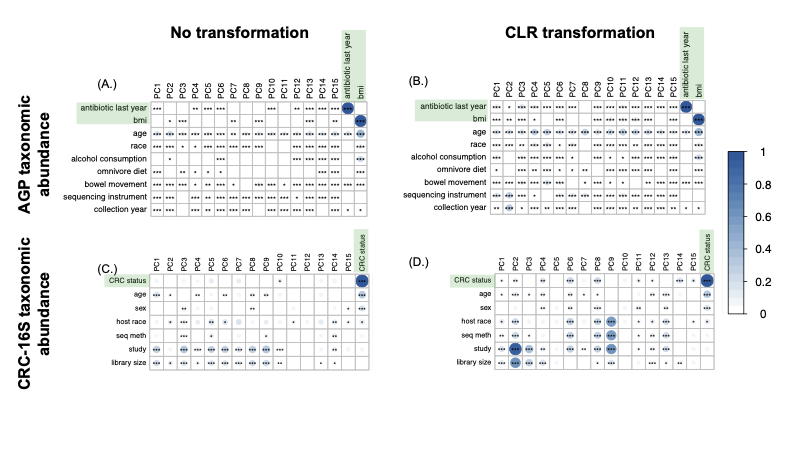

Supplement: S2 Fig — The first 15 PCs in the CRC-16S taxonomic abundance joined datasets are correlated with variables measured in each of the studies, including phenotype, sex, age, race, dataset label, sequencing method, library size and several others in (A, B) AGP, (C, D) CRC-16S. The size and color of the circles in each cell indicate the magnitude of correlation while black asterisks indicate the significance of the Pearson correlation of the PCs with each of the variables. The color bar at right of each plot represents the range of correlations observed across all datasets. [*,**,*** indicate p-values as follows: 10−2 < p < 0.05, 10−3 < p < 10−2, p < 10−3]. (TIF) [file pcbi.1009838.s002.tif]

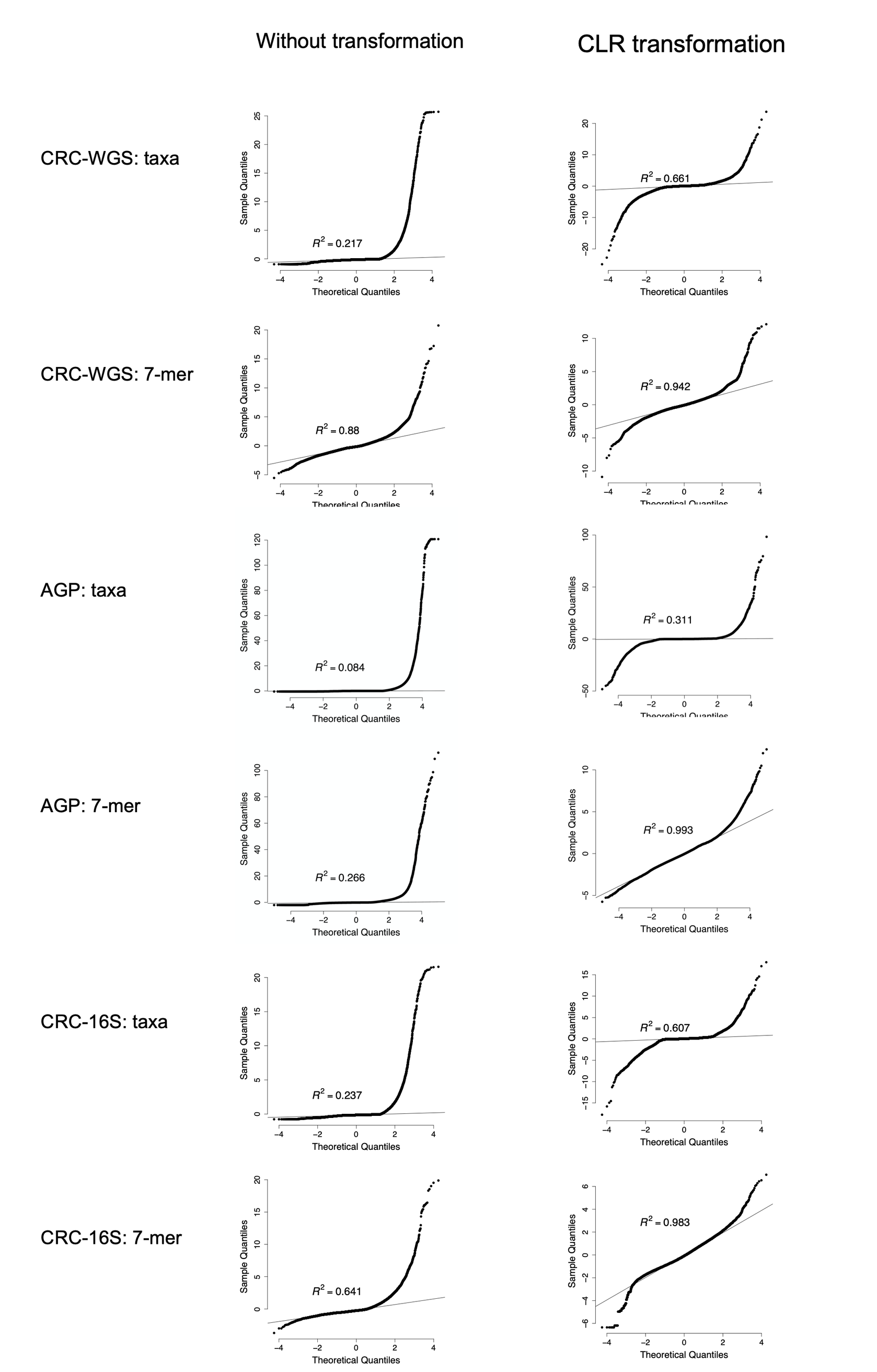

Supplement: S3 Fig — The quantiles of 100 randomly-selected taxonomic features or k-mers, that were converted to z-scores, ranked against the expected quantiles from a normal distribution of mean 0 and variance 1. The R-squared values are reported in the annotated text. (TIF) [file pcbi.1009838.s003.tif]

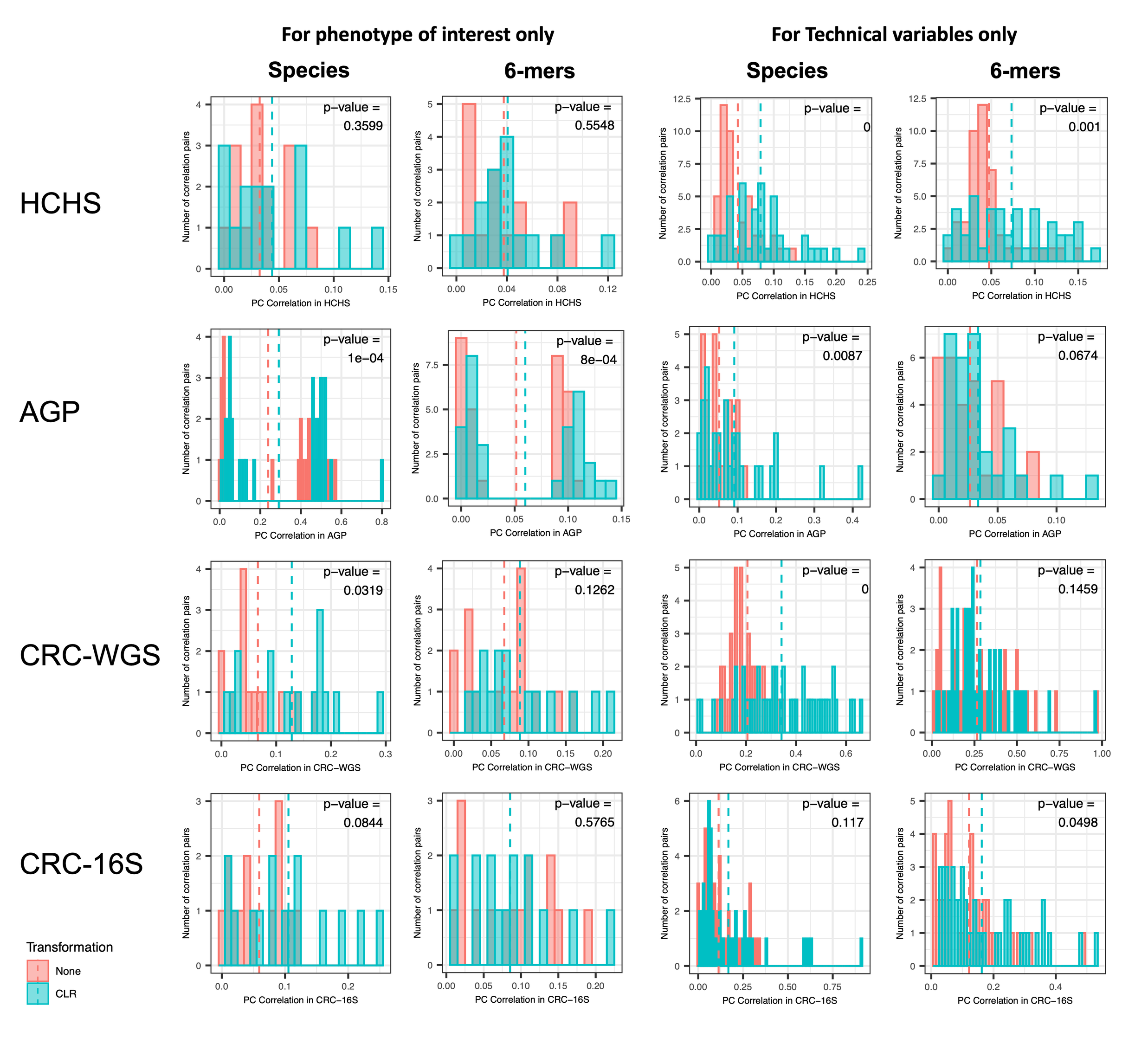

Supplement: S4 Fig — Histograms show the distribution of correlation values computed between the top 15 PCs of taxonomic features in each dataset and the phenotype covariates and technical covariates. Shown in black text are the Kolmogorov-Smirnov test p-values for the test of the null hypothesis that the distribution of correlations in the non-transformed data is no different from the correlations in the CLR-transformed data. HCHS is the only dataset with significant increase in correlation in the technical covariates but not the phenotype of interest. (TIF) [file pcbi.1009838.s004.tif]

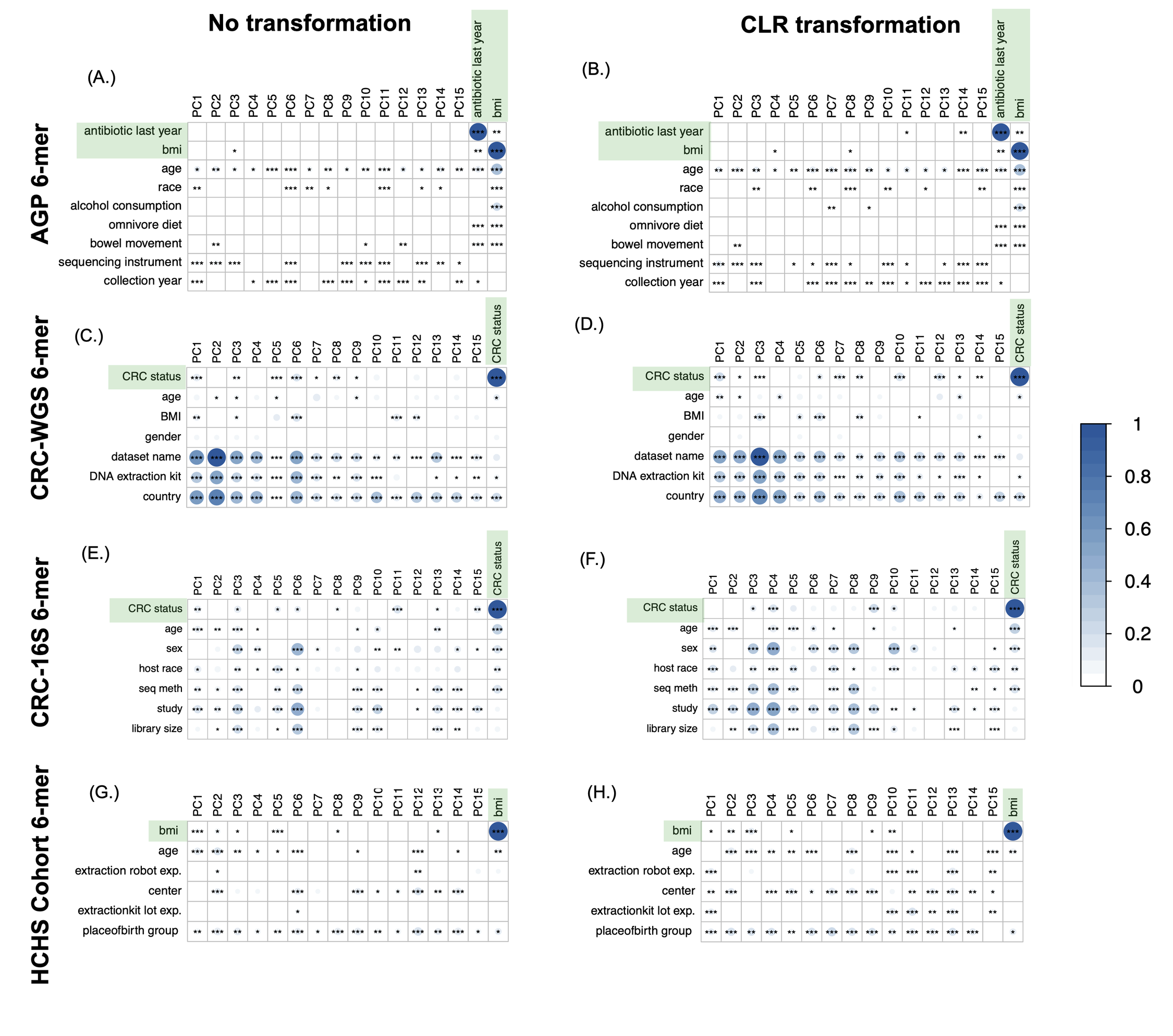

Supplement: S5 Fig — The first 15 PCs before (a, c, e, and g) and after (b, d, f, and h) the CLR-transformation are correlated with variables measured in each of the studies, including dataset label, library size, DNA extraction kit used, country of origin, age, body mass index (BMI), sex, and colorectal cancer status (CRC). The size and color of the circles in each cell indicate the magnitude of correlation while black asterisks indicate the significance of the Pearson correlation of the PCs with each of the variables. The color bar at right of each plot represents the range of correlations observed across all datasets. [*,**,*** indicate p-values as follows: 10−2 < p < 0.05, 10−3 < p < 10−2, p < 10−3]. (TIF) [file pcbi.1009838.s005.tif]

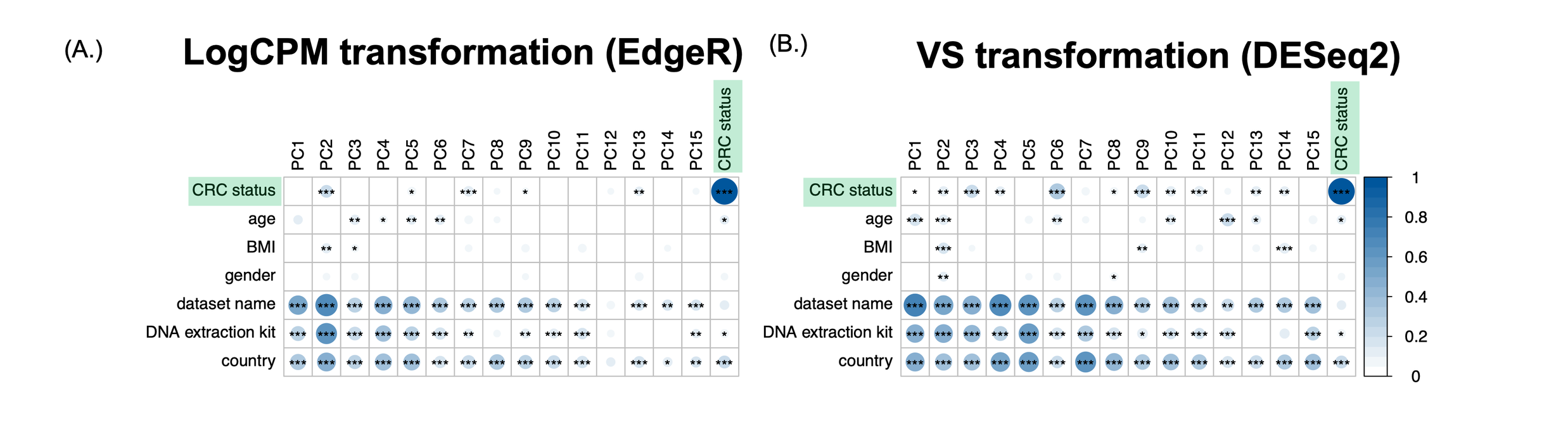

Supplement: S6 Fig — The first 15 PCs from data transformed with the (A) EdgeR log counts per million (LogCPM) transformation[36] and (B) DESeq2 Variance Stabilizing (VS) transformation are correlated with variables measured in each of the studies, including dataset label, library size, DNA extraction kit used, country of origin, age, body mass index (BMI), sex, and colorectal cancer status (CRC). The size and color of the circles in each cell indicate the magnitude of correlation while black asterisks indicate the significance of the Pearson correlation of the PCs with each of the variables. The color bar at right of each plot represents the range of correlations observed across all datasets. [*,**,*** indicate p-values as follows: 10−2 < p < 0.05, 10−3 < p < 10−2, p < 10−3]. (TIF) [file pcbi.1009838.s006.tif]

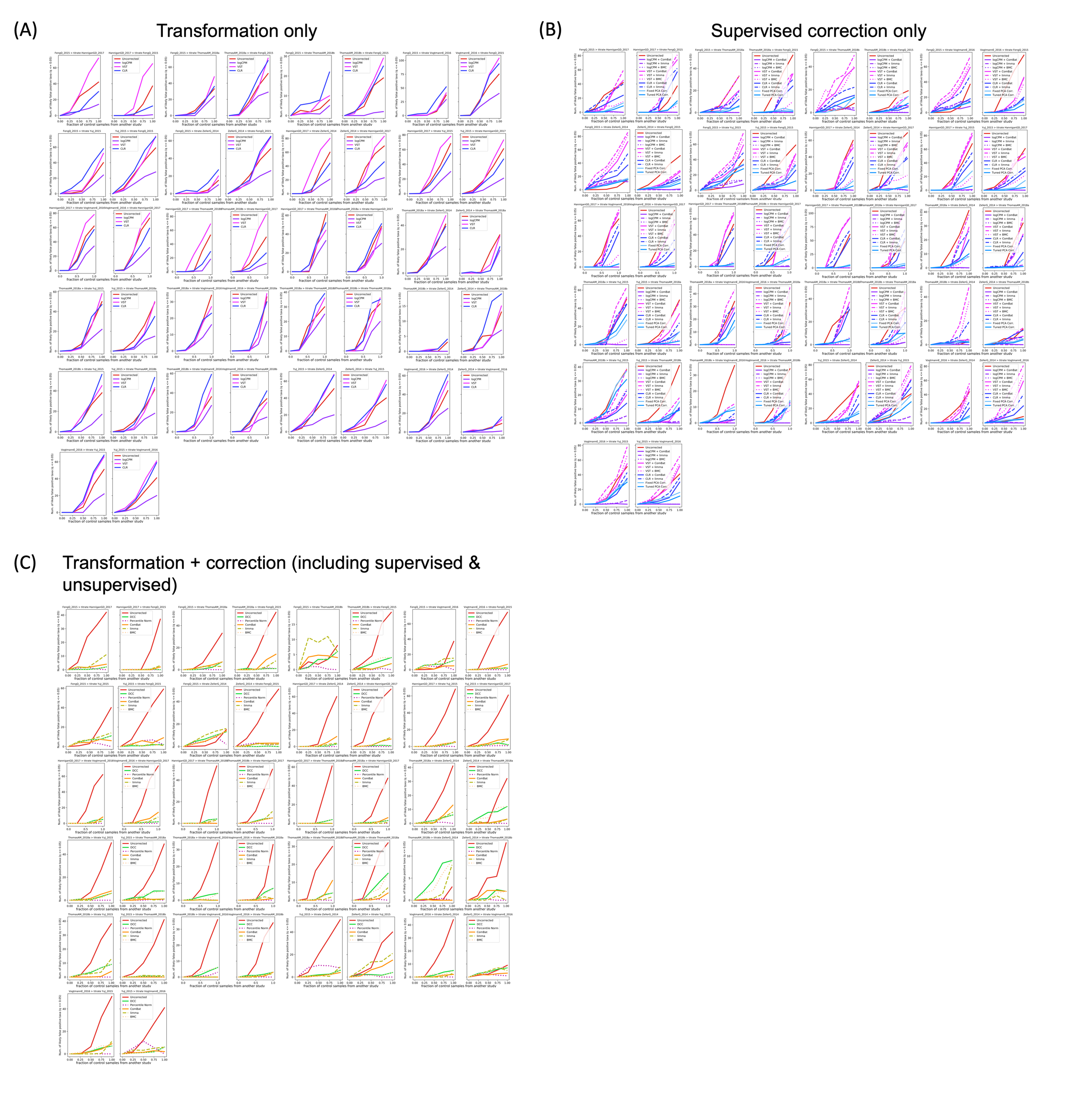

Supplement: S7 Fig — For each study in CRC-WGS, an equal number of cases and controls were drawn to determine significant taxa associated with CRC. Then, at proportions of 25%, 50% and 100%, control samples were replaced with controls from a second study. This experiment was repeated after applying (A) transformations, (B) corrections, or (C) a combination of both (including unsupervised methods) to compare the extent to which new false positive associations arise with increasing confounding between CRC and study label. (TIF) [file pcbi.1009838.s007.tif]

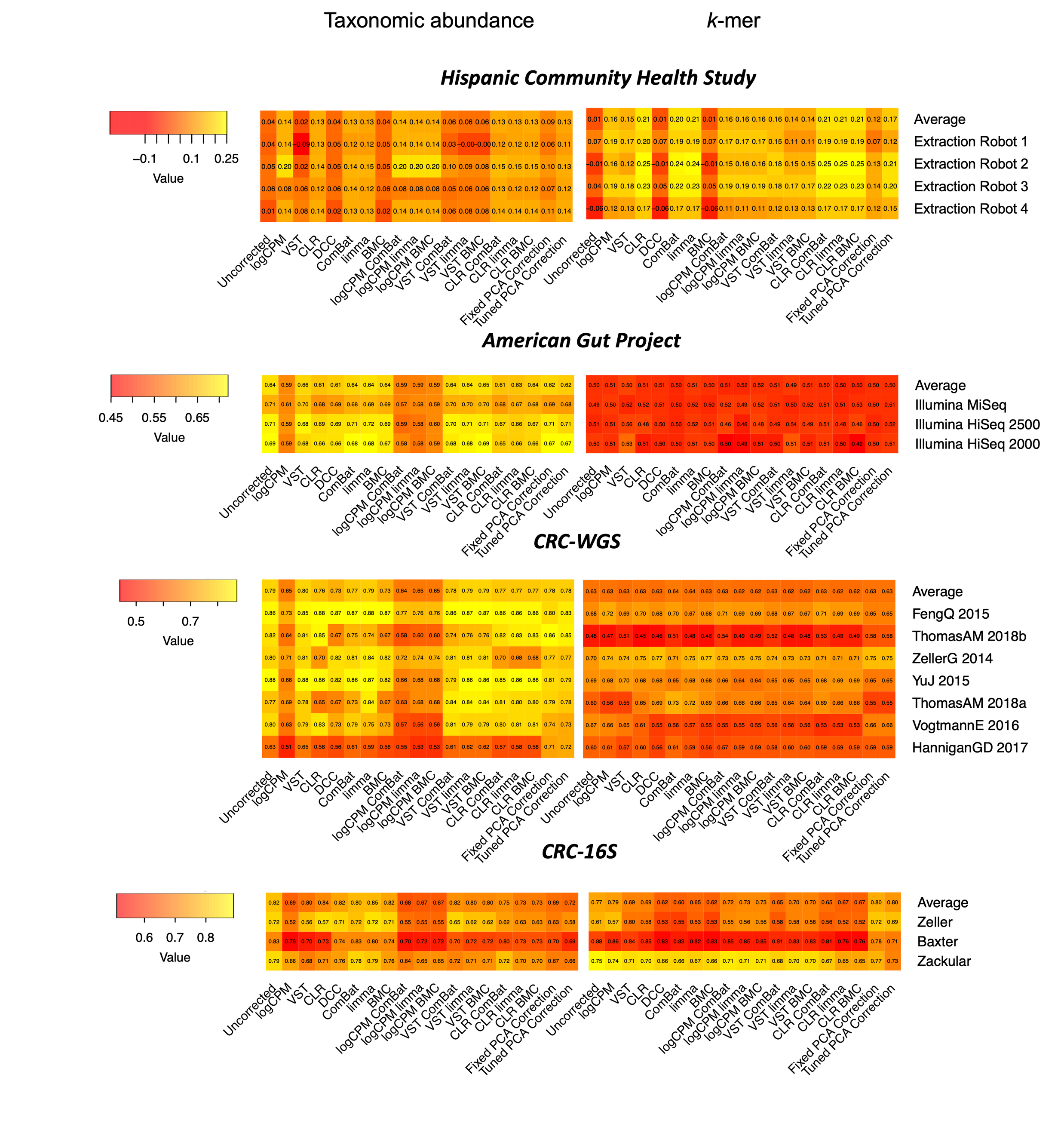

Supplement: S8 Fig — Heatmap showing AUC or Pearson correlation in a cross-validated prediction model using either uncorrected data or data after applying one of the following covariate correction approaches: DCC, ComBat[64], limma[63], BMC[62], and Fixed PCA correction with three PCs regressed out, and Tuned PCA correction where the number of PCs regressed out is a tuned hyperparameter. The testing accuracy mean shown is obtained from a five-fold cross validation repeated 10 times. (TIF) [file pcbi.1009838.s008.tif]
