# Supplementary material for: Evaluating supervised and unsupervised background noise correction in human gut microbiome data
Source: PLoS Comput Biol. 2022 Feb 7;18(2):e1009838. doi: 10.1371/journal.pcbi.1009838 (PMC8853548; doi:10.1371/journal.pcbi.1009838)
Supplement: S1 Table — Shown is the mean number of likely false positive associations with respect to the original study 1 case and controls before adding control samples from study two, across all pairs of studies within CRC-WGS and across all five-fold replicates of titration at each mixing proportion of 0%, 25%, 50%, 75%, and 100% controls from study two. (DOCX) [file pcbi.1009838.s009.docx]

|  |  | **Proportion of controls from second study** | | | | |
| --- | --- | --- | --- | --- | --- | --- |
|  |  | **0** | **0.25** | **0.5** | **0.75** | **1** |
|  |  |  |  |  |  |  |
| Data Transformation | **Uncorrected** | 0 | 1 | 10 | 26 | 42 |
|  | **logCPM** | 0 | 1 | 4 | 12 | 20 |
|  | **VST** | 0 | 1 | 12 | 32 | 52 |
|  | **CLR** | 0 | 2 | 10 | 28 | 44 |
| Supervised Correction | **DCC** | 0 | 1 | 2 | 4 | 5 |
|  | **Percentile normalization** | 0 | 1 | 1 | 1 | 0 |
|  | **ComBat** | 0 | 1 | 1 | 3 | 5 |
|  | **limma** | 0 | 1 | 2 | 3 | 6 |
|  | **BMC** | 0 | 1 | 2 | 3 | 5 |
| Transformation + Correction (including unsupervised) | **logCPM + ComBat** | 0 | 0 | 1 | 1 | 1 |
|  | **logCPM + limma** | 0 | 1 | 1 | 1 | 2 |
|  | **logCPM + BMC** | 0 | 1 | 1 | 1 | 2 |
|  | **VST + ComBat** | 0 | 2 | 7 | 25 | 44 |
|  | **VST + limma** | 0 | 3 | 10 | 32 | 55 |
|  | **VST + BMC** | 0 | 3 | 5 | 11 | 25 |
|  | **CLR + ComBat** | 0 | 2 | 4 | 13 | 26 |
|  | **CLR + limma** | 0 | 3 | 6 | 20 | 35 |
|  | **CLR + BMC** | 0 | 16 | 36 | 94 | 173 |
|  | **Fixed PCA correction** | 0 | 1 | 3 | 7 | 14 |
|  | **Tuned PCA correction** | 0 | 1 | 2 | 6 | 11 |
